# Association between aromatic amines and serum neurofilament light chain as a biomarker of neural damage: a cross-sectional study from NHANES

**DOI:** 10.3389/fpubh.2024.1344087

**Published:** 2024-09-24

**Authors:** Tong Lin, Haiyan Mao, Shanshan Huang, Jialu Chen

**Affiliations:** Department of Critical Medicine, Ningbo Medical Center Lihuili Hospital, Ningbo, China

**Keywords:** aromatic amines, 2-Aminonaphthalene, serum neurofilament light chain, NHANES, nerve injury

## Abstract

**Background:**

Aromatic amines (AAs) are a group of compounds widely found in chemical industry, tobacco smoke, and during food processing, with established carcinogenic properties. To date, there have been no reports on the potential neurotoxic effects of adult exposure to AAs. Serum neurofilament light chain (sNfL) is a protein released into the bloodstream following nerve axon injury and has been validated as a reliable biomarker for various neurological diseases. However, there has been no research to investigate the relationship between AAs exposure and sNfL.

**Methods:**

In this study, we selected adults (aged ≥20 years) with data on both AAs and sNfL from the National Health and Nutrition Examination Survey (NHANES) conducted in 2013–2014. We used multivariable linear regression models to explore the correlation between urinary AAs and sNfL.

**Results:**

In total, 510 adult participants with an average age of 43.58 ± 14.74 years were included in the study. Our findings indicate that, based on univariate linear regression and between-group comparative analyses, 1-Aminonaphthalene (1-AN), 2-Aminonaphthalene (2-AN), 4-Aminobiphenyl (4-AN) and o-Anisidine (o-ANI) showed a positive correlation with serum neurofilament light chain (*P* < 0.05). However, multiple linear regression analysis revealed that only 2-AN exhibited a positive correlation with serum neurofilament light chain (*P* < 0.05), while the correlations of other compounds with serum neurofilament light chain became non-significant.

**Conclusion:**

Although our cross-sectional study fails to establish causal relationships or determine clinical significance, the findings indicate a potential association between adult exposure to AAs, notably 2-AN, and nerve damage. Consequently, further research is needed to explore the connection between AAs exposure, sNfL, and neurological conditions in adults.

## Background

Aromatic amines (AAs) are a category of organic compounds characterized by the presence of a benzene ring and amino functional groups. These compounds are widely found in various industrial products, including dyes and pigments (such as azo dyes and indigo dyes), pharmaceuticals, pesticides, herbicides, synthetic rubber, plastics (1) and even be released during the cooking process of edible oils (2). Aromatic amines can enter the human body through inhalation, skin contact, or ingestion.

However, a significant concern associated with aromatic amines is their presence in tobacco smoke. Tobacco smoke is a complex mixture containing thousands of compounds, including over 60 known carcinogens, some of which are aromatic amines (3). People can be exposed to aromatic amines by inhaling tobacco smoke, using tobacco-related products (such as smoking or chewing tobacco), or being exposed to secondhand smoke (the smoke released by others who are smoking) (4). The U.S. Food and Drug Administration (FDA) has established a roster of harmful and potentially harmful constituents (HPHCs) found in tobacco products and tobacco smoke. Among these, aromatic amines, including 1-aminonaphthalene (1-AN), 2-aminonaphthalene (2-AN), and 4-aminobiphenyl (4-AN), have been included on the FDA’s HPHC list (5). Moreover, the International Agency for Research on Cancer (IARC) has categorized 2-aminonaphthalene, 4-aminobiphenyl, and o-toluidine (o-TOL) as Group 1 carcinogens due to their association with the development of various malignant tumors (6). Aromatic amines are primarily metabolized in the liver, subsequently entering the bladder and ultimately being excreted through urine. Extensive research has established the genotoxic mechanisms of aromatic amines, particularly in relation to DNA adduct formation and mutagenesis (7). Occupational exposure to aromatic amines has been demonstrated to significantly elevate the risk of bladder cancer (8, 9). Furthermore, certain animal experiments have suggested that 2-acetylaminofluorene, a constituent of aromatic amines, can instigate hepatocarcinogenesis in mice (10).

Serum neurofilament light chain (sNfL) is a major cytoskeletal protein of neuronal axons, often referred to as one of the subunits of neuronal neurofilaments (NF), which are structural proteins in neurons playing a crucial role in maintaining the morphology and function of neurons; sNfL, being a constituent of NFs, is typically released into the bloodstream in only minimal amounts under normal physiological conditions (11). However, when neurons are subjected to damage or in cases of neurodegenerative diseases that result in neuronal injury, there is a significant increase in the release of sNfL. Therefore, measuring sNfL levels in serum can serve as a non-specific biomarker for neuronal injury. This has significant value in the diagnosis, disease progression monitoring, and prognosis assessment of neurological conditions such as neurodegenerative diseases [e.g., Alzheimer’s disease (12), Parkinson’s disease (13), multiple sclerosis (14), ischemic stroke (15)]. Elevated serum sNfL levels can reflect the extent and progression of neuronal damage, making it useful for assessing disease severity, predicting the trajectory of the disease, and monitoring treatment efficacy (16).

Regrettably, there is currently no existing research examining the connection between AAs levels and adult neurological function. To address this gap, we have obtained accessible data on urinary AAs metabolites and serum sNfL from the 2013–2014 National Health and Nutrition Examination Survey (NHANES). The primary objective of this study is to investigate the potential relationship between AAs exposure and serum sNfL levels within a demographically representative sample of the U.S. population.

## Materials and methods

### Ethical statement

The NHANES protocol received authorization from the National Center for Health Statistics (NCHS) of the Centers for Disease Control and Prevention (CDC), US and written informed consent was obtained from all participants upon admission. The Ethics Review Committee of Ningbo Medical Center Lihuil Hospital concluded that the study was exempt from ethical review since it utilized de-identified data that was publicly accessible.

### Study population

**Figure 1:**
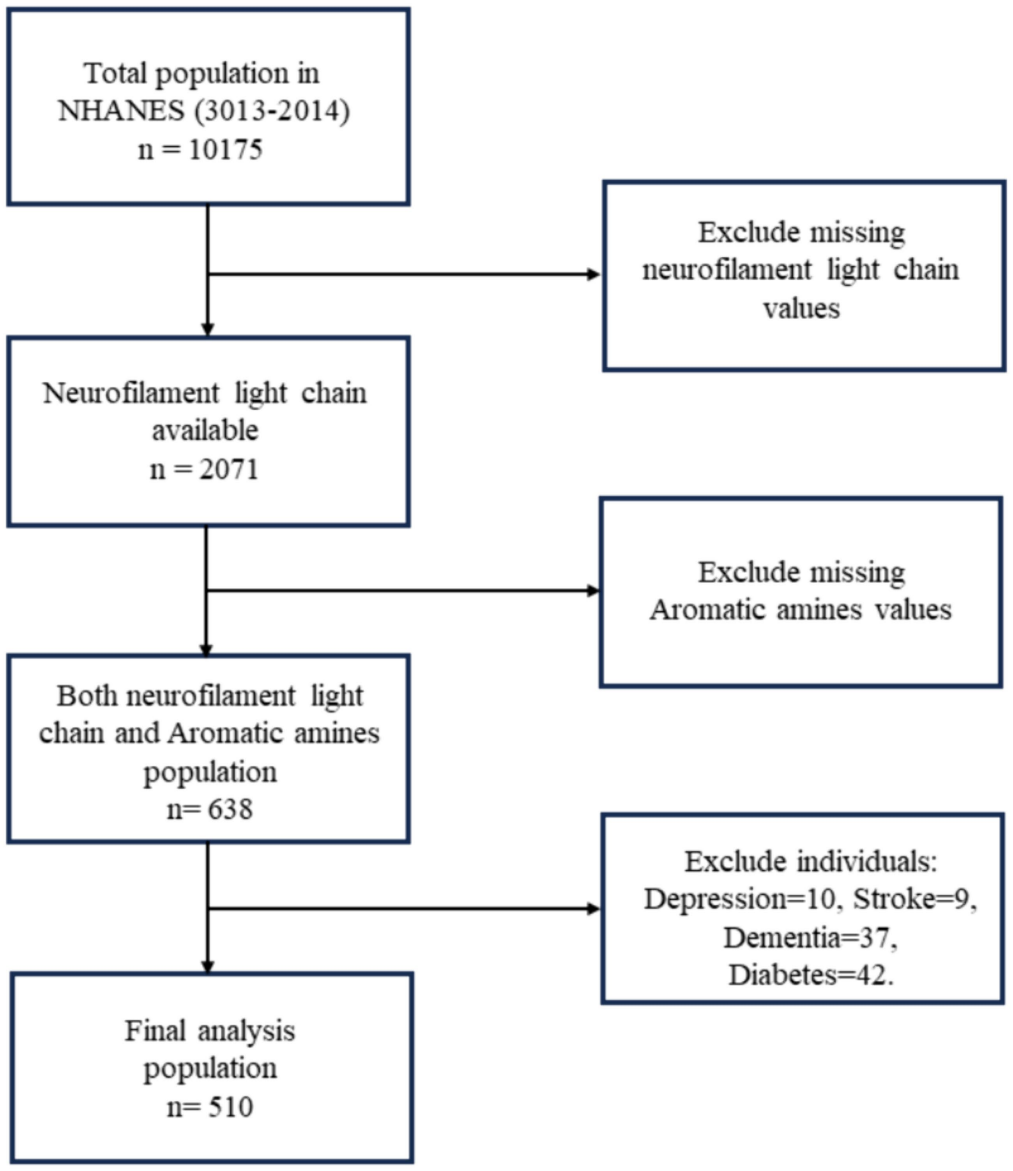
Flowchart algorithm for study population inclusion (National Health and Nutrition Examination Survey, NHANES).

The National Health and Nutrition Examination Survey (NHANES) in the United States is a nationwide survey that recruits a representative sample of citizens every 2 years. Detailed survey methods and participant consent forms can be found on the NHANES website (https://wwwn.cdc.gov/nchs/nhanes/). In NHANES 2013–2014, a total of 10,175 participants were involved. Urinary AAs metabolites were measured in participants aged 6 years and older. sNfL were assessed in participants aged 20 to 75 years, with a total of 2,071 valid samples. Data is available for 510 study subjects for variables common to both datasets, excluding those with diseases known to cause elevated sNfL levels, such as depression (17), dementia (18), stroke (19), and diabetes (20), which were identified through the NHANES questionnaire. Depressive symptoms were measured using the Patient Health Questionnaire-9 (PHQ-9), with a score of ≥10 indicating depression. Dementia was identified through cognitive function tests, and stroke was determined via a questionnaire. The workflow is illustrated in Figure 1.

### Measurement of urine aromatic amines metabolites

NHANES 2013–2014 database contains six urinary aromatic amines metabolites: 1-Aminonaphthalene (1-AN), 2-Aminonaphthalene (2-AN), 4-Aminobiphenyl (4-AN), o-Anisidine (o-ANI), 2,6-Dimethylaniline (2,6-Dim), and o-Toluidine (o-Tol). Aromatic amines are quantified using a precise method involving isotope-dilution gas chromatography and tandem mass spectrometry (ID GC–MS/MS). Urine samples are collected and stored at approximately −70°C. Internal standards are added, and the samples undergo hydrolysis, cleanup, and extraction. Analytes are then derivatized and analyzed by GC/MS/MS with multiple reaction monitoring (MRM). Details regarding the urinary aromatic amines metabolites detection method can be accessed on the homepage (https://wwwn.cdc.gov/Nchs/Nhanes/2013-2014/AA_H.htm).

### Measurement of sNfL

In NHANES 2013–2014, serum sNfL levels were measured using a highly sensitive immunoassay system developed by Siemens Healthineers. The analytical method utilizes acridinium ester (AE) chemiluminescence and paramagnetic particles on the Attelica platform. This involves incubating the sample with AE-labeled antibodies that bind to the sNfL antigen. Paramagnetic particles coated with capture antibodies are then added to form antigen–antibody complexes. After removing unbound AE-labeled antibodies, chemiluminescence is initiated with acid and base, and light emission is measured. This process is fully automated on the Attelica immunoassay system. Details regarding the urinary aromatic amines metabolites detection method can be accessed on the homepage (https://wwwn.cdc.gov/Nchs/Nhanes/2013-2014/SSSNFL_H.htm).

### Covariates

We systematically examined a wide array of demographic attributes, encompassing age, gender, race/ethnicity, educational level, smoking habits, alcohol consumption, and a medical history encompassing hypertension. These extensive data points were collected through structured family interviews utilizing standardized questionnaires. In terms of race/ethnicity, participants were categorized into groups recommended by NHANES, which included Mexican American, other Hispanic, non-Hispanic white, non-Hispanic black, and other races (including multi-racial). Educational attainment was classified into three levels: below high school, high school, and above high school. Smoking status was defined as having smoked at least 100 cigarettes during one’s lifetime. Alcohol consumption was determined by a positive response to the question, “In any 1 year, have you had at least 12 drinks of any type of alcoholic beverage?” Body mass index (BMI) was calculated as weight in kilograms divided by height in meters squared (kg/m^2^) during the physical examination. Biochemical measures, including alanine aminotransferase (ALT), blood lead levels in the blood sample, were quantified through laboratory analysis and estimated glomerular filtration rate (eGFR) (21) calculated from serum creatinine.

### Statistical analysis

Due to the non-normal distribution of aromatic amine metabolites and sNfL levels, natural logarithms of these two variables were employed in our analyses. Sampling weights were used based on the methods recommended on the NHANES website. Continuous variables are presented as means [standard deviation (SD)] or medians (interquartile range, IQR), while categorical variables are presented as frequencies or percentages (*n*, %). Chi-squared tests were used to analyze categorical variables, and Student’s two-tailed t test or Mann–Whitney U tests were used to assess descriptive variable differences between different genders. In the general linear model for our sample (model 1), sNfL was considered the dependent variable, and aromatic amine metabolites were treated as independent variables, categorized into three groups for inter-group comparisons. In multivariate linear regression models, an extended model approach was employed for covariate adjustments. Model 2 included age, gender, ethnicity, BMI, smoking, and drinking status. Model 3 further adjusted for the presence of chronic conditions such as hypertension. Model 4, built on model 3, additionally incorporated biochemical indicators. Subsequent to the statistical significance observed in models 2 and 3 for the metabolite 2-AN, subgroup analyses and discussions were conducted.

All analyses were conducted using R 4.0.5 statistical software (The R Foundation) and GraphPad Prism 8.0. A two-sided value of *p* < 0.05 was considered statistically significant.

## Results

### Participant characteristics

A total of 510 adults were included (mean age 43.58 ± 14.74 years), with 256 (50.20%) being female. Participant characteristics are summarized by gender in Table 1. The average ages of males and females were 42.97 ± 14.79 years and 44.18 ± 14.69 years, respectively. In comparison to the male group, there were no significant differences in education level, ethnicity, or the presence of chronic conditions hypertension in the female group (*p* > 0.05). Notably, the female group had a higher BMI (29.21 ± 7.32) compared to the male group (27.58 ± 6.28), and the proportion of alcohol intake and smoking was higher in males (87.93% vs. Sixty five.22 and 51.57% vs. 29.30%, respectively). Additionally, there were differences in blood lead and liver function between the male and female groups (*p* < 0.05). sNfL levels and urinary levels of 1-AN, 2-AN and o-ANI were higher in the male group (*p* < 0.05).

**Table 1:** Baseline characteristics of the study population by gender.

Variables	Total (*n* = 510)	Male (*n* = 254)	Female (*n* = 256)	*p*-value
Age	43.58 ± 14.74	42.97 ± 14.79	44.18 ± 14.69	0.354
Education				0.145
Less than 9th grade	32 (6.27)	20 (7.87)	12 (4.69)	
9-11th grade	63 (12.35)	33 (12.99)	30 (11.72)	
High school graduate	105 (20.59)	60 (23.62)	45 (17.58)	
Some college or AA degree	168 (32.94)	77 (30.31)	91 (35.55)	
College graduate or above	142 (27.84)	64 (25.20)	78 (30.47)	
Race				0.871
Mexican American	82 (16.08)	40 (15.75)	42 (16.41)	
Other Hispanic	51 (10.00)	22 (8.66)	29 (11.33)	
Non-Hispanic White	229 (44.90)	118 (46.46)	111 (43.36)	
Non-Hispanic Black	77 (15.10)	38 (14.96)	39 (15.23)	
Others	71 (13.92)	36 (14.17)	35 (13.67)	
Hypertension	136 (26.67)	64 (25.20)	72 (28.12)	0.455
Drink	354 (76.62)	204 (87.93)	150 (65.22)	0.000
Smoke	206 (40.39)	131 (51.57)	75 (29.30)	0.000
BMI (kg/m^2^)	28.39 ± 6.86	27.58 ± 6.28	29.21 ± 7.32	0.007
Blood lead (ug/dL)	0.95 (0.61, 1.47)	1.14 (0.73, 1.68)	0.77 (0.51, 1.26)	0.000
ALT (U/L)	21.00 (17.00, 28.00)	24.50 (19.25, 31.00)	18.00 (14.00, 23.00)	0.000
eGFR (mL/min/1.73 m2)	113.53 (90.30, 138.00)	116.25 (96.23, 139.46)	108.57 (84.10, 136.37)	0.089
ln sNfL	2.38 ± 0.64	2.48 ± 0.64	2.28 ± 0.62	0.000
ln 1-AN	1.34 ± 1.61	1.57 ± 1.68	1.10 ± 1.51	0.001
ln 2-AN	1.75 ± 0.97	1.90 ± 1.01	1.61 ± 0.90	0.000
ln 4-AN	1.82 ± 1.22	1.93 ± 1.20	1.72 ± 1.24	0.057
ln o-ANI	3.53 ± 0.81	3.68 ± 0.83	3.39 ± 0.77	0.000
ln 2,6-Dim	3.36 ± 1.35	3.44 ± 1.38	3.29 ± 1.32	0.235
ln o-Tol	5.73 ± 0.90	5.78 ± 0.89	5.67 ± 0.91	0.157

Continuous variables are expressed as mean (SD) or median (interquartile range), and categorical variables are expressed as *n* (%). BMI, body mass index; ALT, alanine aminotransferase; eGFR, estimated glomerular filtration rate; 1-AN, 1-Aminonaphthalene, 2-AN, 2-Aminonaphthalene, 4-AN, 4-Aminobiphenyl, o-ANI, o-Anisidine; 2,6-Dim, 2,6-Dimethylaniline; o-Tol, o-Toluidine. The results of sNfL and AAs were subjected to natural logarithm transformation. ln, Napierian Logarithm.

### Univariate linear regression analyses

**Figure 2:**
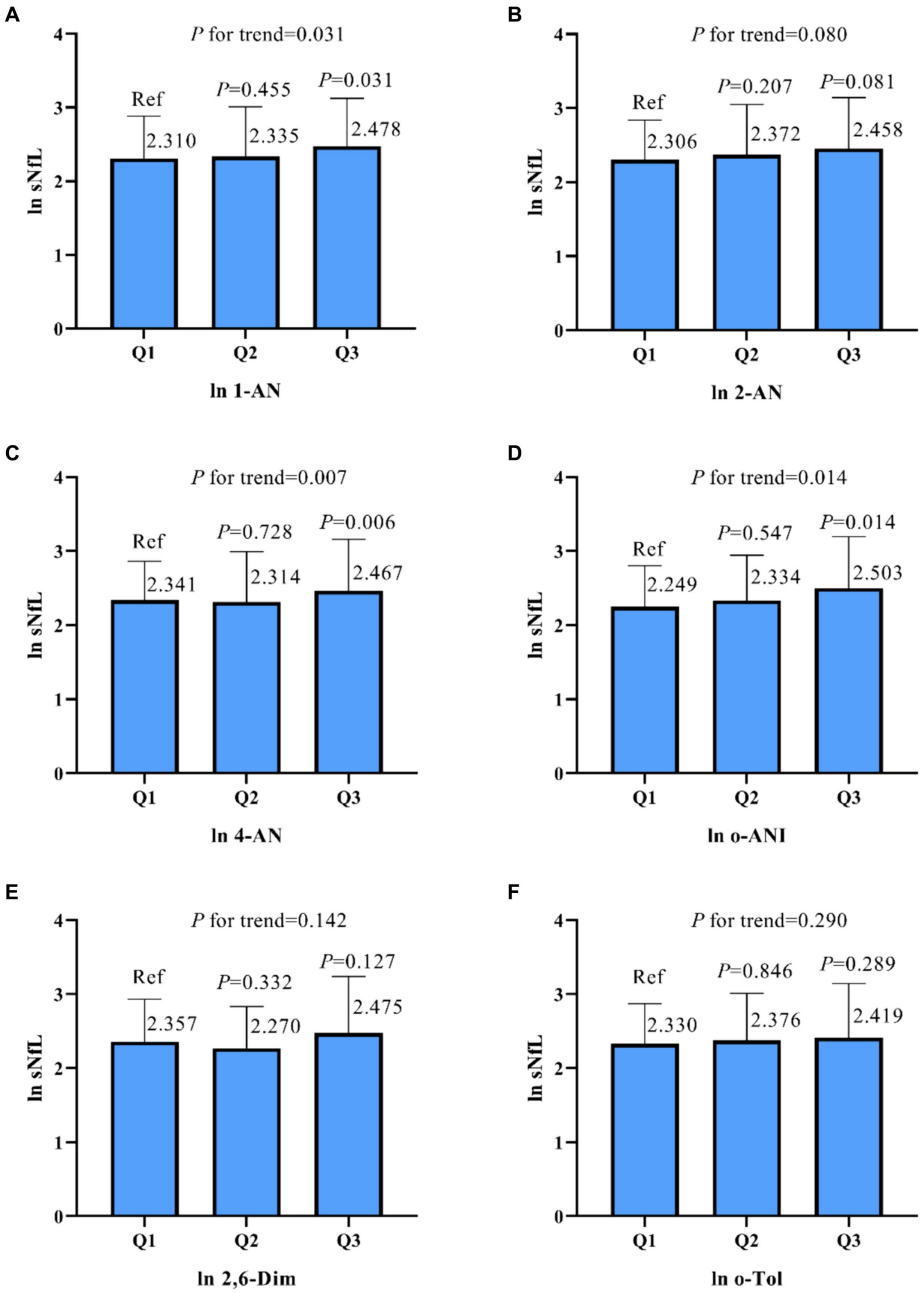
Based on the results of a univariate linear regression, urine samples were classified into three groups based on the concentration of aromatic amines (AAs), and the relationship between the concentration in these groups and the neurofilament light chain (sNfL) was compared. The results for sNfL and AAs were subjected to natural logarithm transformation, and the analysis was conducted using a weighted strategy. Panel **(A)** represents the association between 1-Aminonaphthalene (1-AN) and sNfL, **(B)** shows the relationship for 2-Aminonaphthalene (2-AN), **(C)** for 4-Aminobiphenyl (4-AN), **(D)** for o-Anisidine (o-ANI), **(E)** for 2,6-Dimethylaniline (2,6-Dim), and **(F)** for o-Toluidine (o-Tol). P-values and trends are indicated for each panel. ln, natural logarithm.

As shown in Table 2, model 1 indicates that in univariate linear regression analysis, there was a positive correlation between sNfL and urinary 1-AN, 2-AN, 4-AN, and o-ANI (*β* values of 0.055, 0.100, 0.052, and 0.147, respectively, *p* < 0.05). Furthermore, the trend analysis of three-class concentration gradients of aromatic amine compounds indicated that as the concentrations of 1-AN, 4-AN, and o-ANI increased, sNfL levels also increased correspondingly (*p* < 0.05, Figure 2).

**Table 2:** Linear regression coefficients (standard error) of ln-serum neurofilament light chain with a unit increase in ln-urine aromatic amine metabolites in multiple linear regression models, with results weighted for sampling strategy.

Types of aromatic amine	ln sNfL							
	Model 1		Model 2		Model 3		Model 4	
	*β* (SE)	*p*-value	Adjusted *β* (SE)	*p*-value	Adjusted *β* (SE)	*p*-value	Adjusted *β* (SE)	*p*-value
ln 1-AN	0.055 (0.016)	0.004	0.045 (0.024)	0.095	0.045 (0.023)	0.086	0.042 (0.025)	0.155
ln 2-AN	0.100 (0.039)	0.023	0.110 (0.036)	0.015	0.105 (0.034)	0.016	0.111 (0.039)	0.038
ln 4-AN	0.052 (0.019)	0.018	0.061 (0.036)	0.130	0.063 (0.036)	0.124	0.043 (0.030)	0.207
ln o-ANI	0.147 (0.049)	0.009	0.097 (0.041)	0.048	0.096 (0.039)	0.004	0.082 (0.046)	0.132
ln 2,6-Dim	0.029 (0.014)	0.055	0.008 (0.014)	0.576	0.011 (0.014)	0.462	0.008 (0.015)	0.629
ln o-Tol	0.028 (0.040)	0.497	0.050 (0.038)	0.228	0.050 (0.037)	0.227	0.038 (0.042)	0.411

Model 1 unadjusted.

Model 2 adjusted for age, gender, ethnicity, BMI, smoking status, drinking status.

Model 3 adjusted for model 2 plus hypertension.

Model 4 adjusted for model 3 plus Blood lead, ALT, eGFR.

### Multivariable linear regression analyses

In model 2, additional variables such as age, gender, ethnicity, BMI, smoking status, and drinking status were included, causing the previously observed correlations of 1-AN and 4-AN with sNfL to disappear. However, 2-AN and o-ANI still exhibited a positive correlation (*p* < 0.05, Table 2). In model 3, after further adjusting for covariate parameters and including the underlying condition of hypertension from model 2, the positive correlation between 2-AN, o-ANI, and sNfL persisted (*p* < 0.05, Table 2). In model 4, after additional adjustments for biochemical indicators (blood lead levels, ALT, and eGFR) based on model 3, 2-AN still showed a positive correlation with sNfL (*p* = 0.038, Table 2).

### Subgroup analysis of 2-AN

Models 1, 2, 3 and 4 consistently showed a positive correlation between 2-AN and sNfL. Subgroup analysis was therefore conducted for 2-AN. The regression coefficient (S.E.) for sNfL per unit increase in ln 2-AN is presented in Table 3. The analysis revealed that as 2-AN level increased, average sNfL levels increased in subgroups of individuals aged 20–40, BMI <25 kg/m^2^, those without hypertension (Table 3).

**Table 3:** Subgroup linear regression analysis of ln 2-AN and ln-serum neurofilament light chain.

Subgroup	Unweighted no./Population size	Adjusted *β* (SE)	*p*-value
**Gender**			
Male	254/28523617	0.078 (0.060)	0.212
Female	256/26683791	0.110 (0.053)	0.055
**Age**			
20–40	221/24218797	0.126 (0.035)	0.003
40–59	200/21989374	0.120 (0.073)	0.121
> = 60	89/8999237	0.068 (0.068)	0.333
**BMI**			
<25	191/19466285	0.126 (0.050)	0.024
25–30	143/17015960	0.032 (0.037)	0.410
> = 30	176/18725163	0.142 (0.068)	0.055
**Hypertension**			
Yes	136/13745973	0.031 (0.087)	0.728
No	374/41461436	0.104 (0.032)	0.006
**Race**			
Non-Hispanic White	229/35251752	0.102 (0.050)	0.060
Others	281/19955657	0.056 (0.051)	0.288

## Discussion

Serum neurofilament light chain (sNfL) is a structural protein of neuronal cells that is released into the bloodstream during neurological damage or disease (11). It is commonly regarded as a biomarker for neuronal injury, and current research indicates its abnormal increase in conditions such as depression (17), dementia (18), stroke (19). Studies have shown a positive correlation between sNfL levels in the general US population and mortality rates (22). Aromatic amine compounds, common in daily life and production, can enter the body through inhalation, skin contact, or ingestion, but their relationship with sNfL has not been reported.

Therefore, we investigated the relationship between aromatic amine compounds and sNfL using data from the NHANES database, excluding individuals with neurological diseases including depression, dementia, stroke to ensure the accuracy of our findings. Additionally, studies have demonstrated significantly elevated levels of serum neurofilament light chain in diabetic patients, a phenomenon relatively uncommon in non-diabetic populations (20). Considering diabetes itself as a potential confounding factor, we excluded diabetic patients from our study. Our research shows a significant correlation between the concentration of the urinary metabolite 2-aminonaphthalene (2-AN) and serum neurofilament light chain (sNfL) levels. While single-factor linear regression showed a positive correlation between several aromatic amine compounds, including 1-AN, 2-AN, 4-AN, and o-ANI, with sNfL, this association disappeared after conducting a multiple-factor linear regression. However, multiple models consistently indicated a statistically significant relationship between 2-AN and serum neurofilament light chain.

In subgroup analysis, the correlation between 2-AN and sNfL appears significant among individuals with hypertension. While direct research reports on the correlation between hypertension and sNfL are lacking, it is plausible that similar underlying factors May be involved. Hypertension could induce biochemical and metabolic alterations that might affect sNfL levels, potentially influencing the association between aromatic amines and sNfL. Nevertheless, further comprehensive research is needed to validate this relationship. Furthermore, in the age group of 20–40, specific factors May contribute to a more pronounced correlation between aromatic amine and neurofilament light chain. These factors include a lower incidence of chronic diseases, a higher risk of exposure to aromatic amines in the work environment, and a higher rate of smoking (23). These factors May lead to prolonged exposure to aromatic amines and consequently result in a significant correlation between 2-AN and sNfL.

This is the first study to establish a link between aromatic amine compounds and biomarkers of adult neurologic injury, suggesting a potential association between aromatic amine compounds and neurological health in adults. Nevertheless, it remains unclear whether elevated sNfL due to 2-AN is clinically significant in terms of neurological diseases. This linear relationship suggests a close connection between the elevation of the metabolic product of aromatic amine 2-AN and the increase in sNfL.

Serum neurofilament light chain is typically used as a biomarker for assessing neurological damage (16). Neurofilaments are the most abundant proteins in mature myelinated axons, comprising light, medium, and heavy chains. Following nerve axon injury, neurofilaments are released into the bloodstream and cerebrospinal fluid (11). Due to their high solubility, sNfL has been recognized as a novel biomarker for several neurologic disorders, including Alzheimer’s disease, Parkinson’s disease, multiple sclerosis and cerebrovascular events (12–15). The levels of sNfL are also associated with the severity of multiple sclerosis and Alzheimer’s disease (24, 25). It is worth noting that there is currently no established threshold level of sNfL that leads to adult neurological injury. Therefore, this elevation May reflect the impact of 2-AN on the nervous system, potentially causing stress or damage to the nervous system, resulting in an increased release of neurofilament light chains. Aromatic amines are substances released in tobacco smoke, and studies have shown that smoking or exposure to secondhand smoke increases the risk of stroke and dementia (26, 27). However, the specific pathogenic mechanism or the substance in tobacco responsible for these effects is not yet clear.

Regrettably, there has been a lack of reported findings regarding the impact or damage to the nervous system associated with aromatic amine compounds. However, we can propose some possible pathological mechanisms. Aromatic amine compounds are primarily metabolized in the liver and then excreted through urine. During the metabolism process, they May form some metabolic products, where the amine functional groups May undergo acetylation or glucuronidation, or they May oxidize into hydroxylamine forms, which can further transform into N-acetyloxy metabolites through acetylation. These N-acetyloxy metabolites May break down, generating highly reactive nitrogen ions, nitroso groups, or free radicals, which can react with large molecules (such as proteins) and DNA in tissues to form adducts (28, 29). These free radicals May play a crucial role in neurological damage and disease. Free radicals are highly reactive molecules that can be generated through various cellular pathways, including oxygen free radicals and nitrogen free radicals. When free radical production is excessive, it May lead to oxidative stress, triggering the pathogenesis of brain injury, ischemia, and other neurological disorders (30, 31).

Furthermore, the characteristics of aromatic amine compounds, such as molecular size, polarity, charge, and lipophilicity, May influence whether they can pass through the blood–brain barrier. Some small and lipophilic aromatic amines May more easily penetrate the blood–brain barrier, which May directly lead to damage to the nervous system (32, 33).

Regarding the metabolism of aromatic amine 2-AN, it is typically carried out by the cytochrome P450 enzyme family. Research has found that 2-aminonaphthalene May significantly reduce the cytochrome P-450 o-demethylation activity in liver microsomes (34). These CYP450 enzymes May have various functions in the nervous system, including influencing the synthesis and degradation of neurotransmitters, thereby affecting neural signal transmission (35). Additionally, CYP450 enzymes May also be involved in the synthesis of neuroprotective substances to combat neurodegenerative diseases (36). For example, the CYP46A1 enzyme (a type of CYP450) can catalyze the hydrolysis of cholesterol in the brain, generating a neuroprotective substance with antioxidant, anti-inflammatory, and apoptosis-regulating properties (37). However, 2-AN may reduce the activity of these enzymes, leading to a weakening of their bioactive functions, resulting in neural dysfunction. These factors May be related to neurological injury and diseases but require further research for clarification.

This study has several limitations. Firstly, the sample size is limited as only NfL data from NHANES 2013–2014 are available, which limits the ability to conduct a comprehensive analysis and increase the credibility of the research results. Secondly, this is a cross-sectional study, so we cannot infer causality. Thirdly, we did not include other pollutants that May co-expose with aromatic amines or potentially significant confounding factors. For instance, heterocyclic aromatic amines (HAAs) are compounds containing nitrogen atoms in a heterocyclic aromatic structure, where an amino group (-NH₂) or substituted amino groups (-NHR, -NR₂) are attached to the aromatic ring. HAAs differ significantly in structure and sources from aromatic amines. They are primarily formed during the high-temperature cooking of meat and can enter the body through ingestion, known for their carcinogenic and neurotoxic effects (38, 39). Although there is currently a lack of research linking HAAs to serum neurofilament light chain levels, their neurotoxicity has been established, which could potentially confound study outcomes. Fourthly, our study population consists of American adults, so we cannot assume that this finding applies to other age groups or other countries.

## Conclusion

While this is a cross-sectional study and causal relationships cannot be inferred, and clinical significance remains uncertain, our study results suggest that exposure to AAs in adults, particularly 2-AN, May potentially lead to nerve damage. Thus, further research is warranted to investigate the relationship between AAs exposure, sNfL, and neurological diseases in adults.

## Data Availability

The original contributions presented in the study are included in the article/Supplementary material, further inquiries can be directed to the corresponding author/s.
